# Systematic review support received and needed by researchers: a survey of libraries supporting Ontario medical schools

**DOI:** 10.29173/jchla29571

**Published:** 2021-12-01

**Authors:** Sandra McKeown, Zuhaib M. Mir, Jennifer A. Ritonja, Eleftherios Soleas

**Affiliations:** 1 Health Sciences Librarian, Bracken Health Sciences Library, Queen’s University, Kingston, ON, Canada; 2 General Surgery Resident, Division of General Surgery, Department of Surgery, Queen’s University, Kingston, ON, Canada; 3 PhD Candidate, Department of Public Health Sciences, Queen’s University, Kingston, ON, Canada; 4 Director of Continuing Professional Development and Adjunct Professor, Office of Professional Development and Educational Scholarship, Queen’s University, Kingston, ON

## Abstract

**Introduction:**

Finding efficient ways to meet the growing demand for library systematic review support is imperative for facilitating the production of high-quality research. The objectives of this study were threefold: 1) to ascertain the systematic review support provided by health sciences libraries at Ontario medical schools and their affiliated hospitals, 2) to determine the perceived educational needs by researchers at these institutions, and 3) to assess the potential usefulness of freely available, online educational modules for researchers that discuss all stages of the systematic review process.

**Methods:**

We conducted a cross-sectional survey in June and July of 2020. Data was analyzed and presented using median and interquartile range (IQR) for continuous measures, and in proportions for categorical measures.

**Results:**

13 of 19 libraries invited provided usable data. Most libraries spent more time supporting systematic reviews via collaboration and participation than by providing educational support. The perceived needs of library users were contrary to the perceived gaps in researcher support provided by the library/institution. All libraries reported they would find freely available, online educational modules useful for training researchers.

**Discussion:**

The next steps for our inter-professional research team will be to develop freely available, online education modules that introduce researchers to all stages of the systematic review process. These modules cannot replace the value that direct support from librarians, biostatisticians or methodology experts can provide, however, they may offer a more efficient way for libraries to familiarize researchers and trainees with best practices and universally accepted reporting guidelines for performing a high-quality review.

## Introduction

Evidence-based medicine relies on systematic reviews as one of the highest levels of research evidence used to guide clinical decision-making [1]. Unlike traditional literature reviews, these evidence syntheses aim to minimize bias by using explicit, systematic methods to address clinical questions about treatment, causation, diagnosis, and prognosis [2]. Systematic reviews can save clinicians from having to individually search for, appraise, and interpret the findings from numerous studies. When conducted properly, they are the most reliable form of evidence to inform clinical decision-making [3].

The production of systematic reviews has been increasing steadily for decades, but many have been conducted and reported poorly, impeding their intended usefulness and value [4, 5]. Another concerning trend is overlapping and discordant reviews on the same topic, which can be confusing for clinicians to navigate while also decreasing the desired time efficiency of seeking out synthesized research in the first place [4]. Furthermore, redundant or poorly done reviews are a form of research waste that should be avoided as much as possible.

Involving librarians in the systematic review process is a best-practice recommendation of international research organizations [2, 6, 7] and has been shown to improve both review methodology and quality of reporting [8–10]. Not surprisingly, the rise in systematic reviews has correlated with an increased demand for both educational support (instruction and research assistance) and collaborative support (participating on review teams) from health sciences librarians in recent years [11-15]. Librarians have reported various barriers and challenges to providing systematic review support, including time constraints, and researchers’ lack of adherence and awareness to established systematic review methodology [11, 13, 16–18]. The methodology challenges reported most frequently among librarians in one study pertained to question formulation (not clear and answerable or defined too narrowly or broadly), study eligibility criteria (not having any), screening for studies (not having at least two researchers screen), and not following established systematic review methods [16]. Research surveys have found that librarians are most likely to contribute to systematic reviews in more traditional roles (e.g., research question formulation, search design and execution, managing results etc.), and less likely to be involved in non-traditional roles (e.g., screening studies, critical appraisal, data extraction etc.) [17, 19], which may result in gaps for researchers seeking support. Indeed, participants of an in-person systematic review searching workshop offered by librarians at the University of Alberta John W. Scott Health Sciences Library indicated that they would like additional workshops on other parts of the systematic review process such as data extraction and statistical analysis [20]. The in-person workshop series pertaining to knowledge syntheses offered by University of Toronto librarians at the Gerstein Science Information Centre also focuses entirely on literature searching [21]. Finding efficient ways to inform researchers about systematic review best practices (for all stages of the review process) may help alleviate librarian time constraints, improve adherence to rigorous methodology, and address gaps that librarians may not be able to meet when providing educational and collaborative support to researchers.

The objectives of this study were: 1) to ascertain the landscape of systematic review support provided by academic and affiliated teaching hospital libraries serving researchers from the six Ontario medical schools, 2) to determine the perceived educational needs and gaps of researchers conducting systematic reviews at these institutions as perceived by the libraries, and 3) to assess the libraries’ perceived usefulness of a freely available, online educational module series for researchers that covers all stages of the systematic review process. Medical schools in Ontario (University of Ottawa, Queen's University, University of Toronto, McMaster University, Western University, and Northern Ontario School of Medicine) represent 13,487 faculty members, 3,673 students registered in Undergraduate Medical Education (Doctor of Medicine – or MD - programs), and 4,379 post-MD trainees, in addition to graduate students enrolled in various Graduate Medical Education Masters and Doctoral programs [22].

## Methods

### Study design and setting

We conducted a cross-sectional survey of libraries between June and July of 2020. Eligible libraries included academic libraries that support the six Ontario medical schools, as well as libraries at affiliated teaching hospitals that may be involved in providing systematic review support. Email invitations with a link to the electronic survey – accompanied by a letter of information and consent – were sent to a total of 19 libraries that support Ontario medical schools (6 academic and 13 hospital libraries). When possible, the heads of these libraries were contacted directly, otherwise, a generic library email address was used. Email recipients were instructed to complete the survey on behalf of their library as a whole and encouraged to discuss the questionnaire with their library team in advance. Email reminders were sent to libraries that had not completed the survey three weeks after the initial invitation was sent. The study was approved by the Health Science and Affiliated Teaching Hospitals Research Ethics Board at Queen’s University.

### Survey tool

Qualtrics online survey software was used to develop and employ the questionnaire, which solicited details about the library demographics, and the type and volume of library support offered for systematic reviews in recent years (see online supplement). The survey questions assessed perceived gaps in systematic review support, as well as perceived educational needs of researchers conducting systematic reviews. Survey participants were also asked about the potential usefulness and uptake of a freely available, online educational module series for researchers covering all stages of the systematic review process. Before deploying the survey, two librarians (one each from a representative academic and hospital library) participated separately in a think aloud exercise with the authors of this study to identify and resolve any issues with the instructions or the survey content and technology [23]. During this exercise over videoconference, the librarians addressed the survey questions sequentially and detailed their thinking, conceptualizations, and factors considered in their responses to a given question. The authors took notes to improve the language throughout to align the librarian’s articulated thinking on a given question with the intended purpose of that item. The result was a more comprehensible questionnaire with aligned content and responses.

### Data analysis

Due to skewness of the data, continuous measures were analyzed using non-parametric methods and presented using median and interquartile range (IQR). For categorical measures, data were presented in proportions. Of the libraries that completed the survey, no response data were missing for our analyses. One hospital-based library reported not providing systematic review support and was therefore excluded from the analysis.

## Results

### Survey respondents

**Fig. 1:**
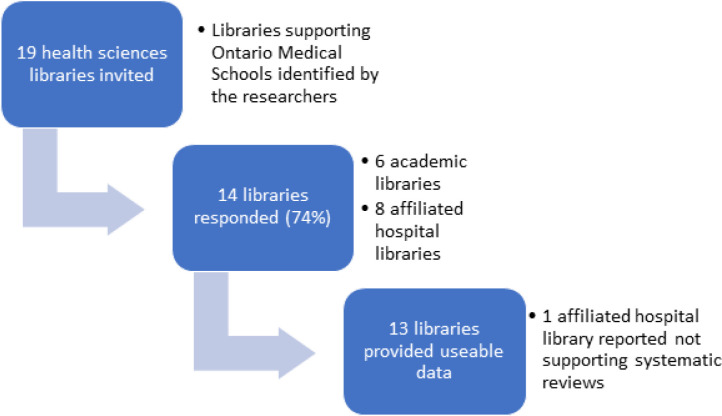
Flow diagram showing library responses invited and analyzed for this study.

In total, 14 of 19 (74%) libraries responded to our survey. All libraries at academic institutions participated (n=6), and 8 libraries in hospital settings participated. One hospital institution reported not providing support for systematic reviews, leaving a total of 13 survey responses for our analyses (figure 1).

### Systematic review support

All 13 libraries reported supporting a variety of user groups for systematic reviews regularly, including faculty, clinicians, trainees and learners (e.g., medical residents, undergraduate students, graduate students, post-doctoral fellows). With the exception of one library, the majority of libraries (12 of 13; 92%) also reported supporting staff users, such as research and educational support professionals.

Users across medical specialties were regularly supported by all 13 libraries. Other disciplines supported during systematic review research included Nursing (n=9), Rehabilitation Therapy (including Physiotherapy and Occupational Therapy) (n=9), Pharmacy (n=6), Life and Health Sciences (n=8), and non-Health Sciences (e.g., Geography, Business, Engineering, Education) (n=3).

In the past two academic years, with the exception of one library, all others provided systematic review support in the form of educational support (i.e., consultations and teaching activities such as workshops or courses) (n=12), and most reported collaboration with or participation on a review team (n=11). Eight libraries also reported providing systematic review support through other means, such as library website content, subject guides, providing access to systematic review software, and collection development. Time spent supporting systematic reviews in educational versus collaborative roles differed greatly between academic and hospital libraries. Among academic libraries, respondents reported spending more of their time on providing educational support (median: 67.0%, IQR: 56.5), followed by collaboration and participation on review teams (median, 33.0%, IQR: 41.5), and then other activities such as tool development (insightScope) (range 0-30%). Conversely, for hospital libraries, respondents reported spending more of their time on collaboration and participation on review teams (median, 80.0%, IQR: 14.3), followed by providing educational support (median: 20.0%, IQR: 16.5). Hospital and academic libraries also differed in terms of authorship on reviews; academic libraries were more likely to report that their librarians were listed as a coauthor when participating on a review team on the majority of reviews (median: 74.0%, IQR: 45), in comparison to hospital libraries (median: 40%, IQR: 77.5).

Overall, when providing educational support, institutions reported that, on average, most of their time (median: 82.5%, IQR: 22.8) was spent on ad-hoc consultations (individual or group), while a much smaller proportion of their time (median: 17.5%, IQR: 24.3), was spent providing formal teaching activities such as library workshops and courses. This finding was similar among hospital and academic libraries.

Ten libraries estimated the number of systematic review projects they supported in an educational or collaborative capacity between 2018 and 2019. Responses ranged from 8 to 285 projects supported in the two-year period, with an average of 112.

### Formal training and perceived needs

Libraries varied in the availability and type of systematic review training provided for researchers at their institutions. Most (n=8) reported their library or institution provided formal workshops, while five reported no formal workshop/guidance being offered at their institution. Four libraries also reported their institution sending researchers to other institutions for systematic review training.

**Fig. 2:**
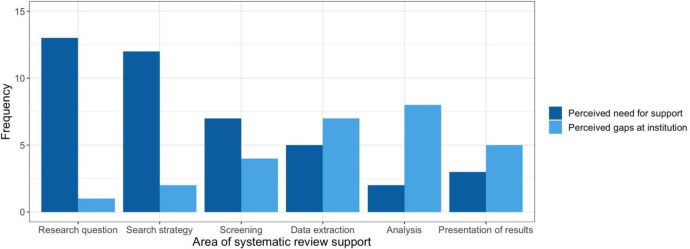
Perceived researcher needs and gaps in systematic review supports.

When assessing aspects of systematic reviews that libraries perceived as requiring the most support by library users, the majority identified developing a research question, search strategy, and screening as areas of needed support (figure 2). Conversely, when asked about perceived gaps in the systematic review support provided by the library/institution, libraries more often identified data extraction, analysis, and presentation of results as being the areas of needed support.

**Fig. 3:**
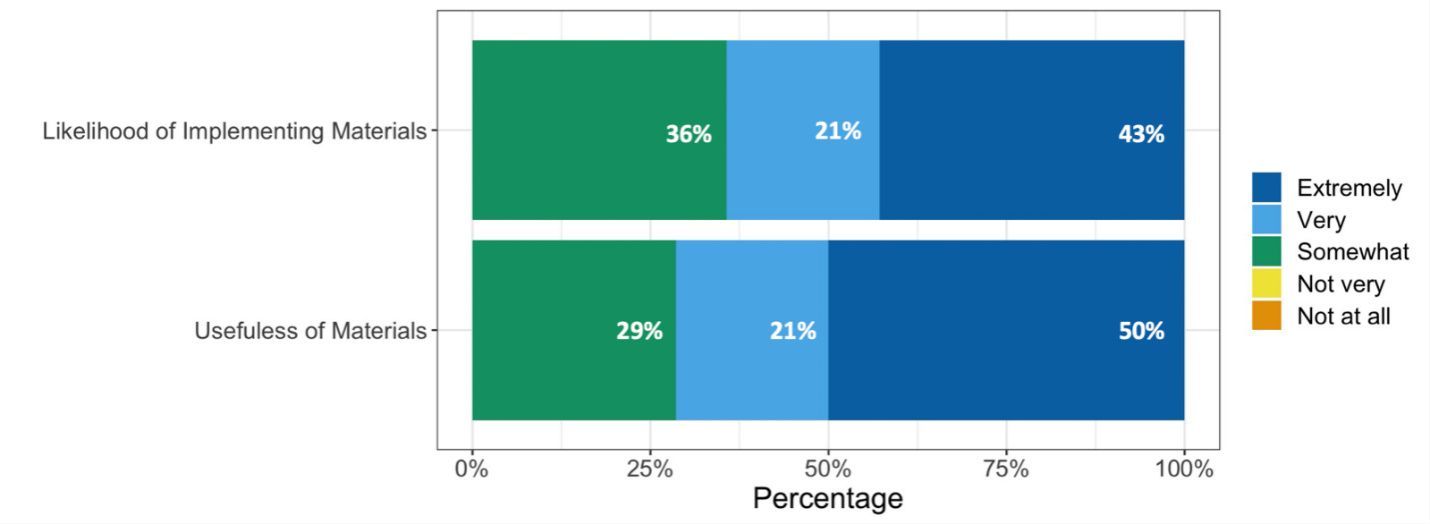
Usefulness and likelihood of implementing or recommending freely available, online educational modules for training researchers.

We asked whether libraries would 1) find prospective, freely available, online educational modules covering all stages of the systematic review process useful for supporting researchers, and 2) be likely to recommend or incorporate these online materials into their current support for researchers conducting systematic reviews. All libraries reported they would find freely available, online educational modules somewhat to extremely useful (figure 3). Similarly, all respondents reported they would be somewhat to extremely likely to incorporate or recommend such content to support researchers (figure 3).

When asked to discuss the reasoning for their responses about the usefulness and likelihood of implementing freely available, online educational modules, two major themes emerged: 1) a need for educational tools to support a growing demand for systematic reviews, and 2) considerations that libraries would like to see for new educational modules. Selected quotes for the two themes are highlighted in Table 1. Five respondents indicated there is an interest and need for more educational materials for systematic reviews. One participant specifically highlighted the need for “an efficient way” to help researchers and would be interested in materials that could be provided to researchers prior to library consultations (table 1). Five respondents also suggested considerations that libraries would like to see for new educational modules. This included the incorporation of materials in other languages (such as French), inclusion of grey literature and discipline-specific topics (e.g. for clinical outcomes), and that the materials meet accessibility standards. One participant also highlighted a barrier to implementation, such that libraries may be less willing to implement educational materials from outside institutions (table 1).

**Table 1:** Reasons provided by libraries for potentially utilizing freely available, online educational modules for researcher training purposes (selected quotes).

Theme 1: *The need for educational resources to support a growing demand for support*	Theme 2: *Considerations for the freely available, online educational modules*
“There is a growing (sic) interest in performing systematic reviews, but a huge lack of understanding as to what they involve. Any help in educating our users would be gratefully appreciated.”	“We would want modular educational tools - small bites that could be sent to a user to address both methodological and technical questions.”
“We are looking for an efficient way of helping researchers. Rather than deliver the same content to each group via consultation we would recommend a course/workshop that they could take before working with us.”	“We also require tools in French, of which there are very few. French language content would be invaluable.”
“There's an interest amongst faculty and learners to better understand what's entailed in conducting knowledge synthesis -type projects.”	“Some of our faculty prefer home-grown resources. It's difficult to know how much support there would be if we recommended something developed by another institution”

## Discussion

An examination of the landscape of systematic review support provided by libraries serving the six Ontario medical schools, and the perceived educational needs of researchers conducting systematic reviews at these institutions, is lacking from the literature. This study provides novel findings from these individual libraries about systematic review support volume and the educational needs and gaps of researchers. Additionally, in looking for efficiencies to help address the increasing demand for library systematic review support, this research survey also gauged potential interest and uptake of prospective, freely available, online educational modules for training researchers.

Academic libraries reported they spent more time on providing educational support rather than collaborative roles over the past two years. Hospital libraries reported an opposite finding, where more time was spent on supporting reviews via collaboration and participation. This suggests that the type of systematic review support offered by libraries differs by the type of institution, which could have important implications for the needs of learners. Still, it is possible that the *number* of faculty, staff and students supported in a purely educational capacity by some of these libraries is greater than the number supported by collaboration and participation; this study did not collect numerical data about *how many* researchers received librarian consultations or attended formal library training sessions in comparison to how many researchers were supported via librarian collaboration and participation.

While library workshops and courses can be an efficient way to educate researchers about systematic reviews, most libraries spent their time providing educational support in the form of ad-hoc consultations (individual or group), a finding that did not differ by type of institution. Librarian consultations may be a very effective method for educating researchers about systematic reviews, as they can be catered to the specific research question, but delivering the same introductory content at each consultation, as one library described it, is not an efficient way of meeting a growing demand for support. It is worth noting that librarian collaboration with or participation on a review team often includes an educational component as well, especially if the researchers have not conducted a systematic review before. Finding efficient ways to meet the educational needs of researchers is therefore relevant to libraries providing one or both forms of systematic review support (educational and collaborative).

The divergence of the perceived needs of library users and the perceived gaps in researcher support at the library and institutional level demonstrates a need for systematic review support that goes beyond the expertise that librarians generally offer. Libraries perceived data analysis as the biggest gap in systematic review support available to researchers, expertise that may be more suitable for biostatisticians or researchers with an epidemiology background to provide. This finding suggests that there is currently insufficient review methodology and biostatistics support available to researchers at Ontario medical schools. If collaboration opportunities with these other methodology experts are not widely available, education modules that cover such content could at least help address this gap by introducing these concepts to researchers.

All library respondents reported that they would find freely available, online educational modules useful in their systematic review support endeavours, and separately that they would be some degree of likely to use or recommend them as an educational resource for researchers. Some of these libraries expressed interest in the ability to share this type of online educational content with researchers to view before they attend the librarian consultation. In this way, online educational modules could cut down on the amount of time librarians spend introducing researchers to the systematic review process and allow them to better maximize their time and expertise during consultations. While there are some courses on conducting systematic reviews in the health sciences already available online, they are generally fee-based [24, 25], or their free versions are designed to take place over the course of several weeks [26, 27].

Libraries surveyed in this study identified a preference for educational resources that meet accessibility standards, allow users to easily navigate content of interest on demand, and that are tailored to stakeholders in a variety of disciplines. In addition to supporting research activities in the field of medicine, most libraries reported supporting the systematic review activities of other disciplines such as nursing, rehabilitation therapy, pharmacy, and other areas of life and health sciences as well. To reach a wider audience of researchers supported by these libraries, the research team plans to develop educational content that builds on research topics and examples that have an inter-disciplinary component. For this reason, the prospective, online educational modules may be useful for health sciences libraries that do not support medical schools as well. Finally, one library respondent also expressed a need for educational resources for French-speaking learners, as few are currently available. Future studies and efforts to ameliorate this need should consider the needs of libraries to provide resources for French-speaking learners.

The next steps for our inter-professional research team will be to develop freely available, online education modules that introduce researchers to all stages of the systematic review process. The proposal for this educational content has already been approved for accreditation by the the Royal College of Physicians and Surgeons Canada and the College of Family Physicians Canada. Physician and surgeon researchers of the Royal College at any Canadian institution, and family physician researchers at any institution in Ontario, would be eligible for continuing education credit after completing the modules. Initially we had planned to offer this systematic review training via in-person workshops at Queen’s University. After the Covid-19 pandemic resulted in librarians and educators having to transition to remote learning for the unforeseeable future, we decided that an online format might be a better option for several additional reasons: asynchronous online modules will allow researchers to access information at the point of need, rather than having to wait for scheduled in-person sessions; online learning provides more flexibility for medical faculty and students, who can otherwise find it difficult to attend in-person workshops (whether full-day or a series offered over multiple days); and covering the stages of the systematic review process in separate modules will allow researchers to learn at their own pace and revisit steps that remain unclear as needed. The online format will also make the content more accessible to learners, as we would have only been able to offer in-person workshops three times per year at most (based on the availability of both librarians and non-librarian instructors). While research into the effectiveness of e-learning for teaching medical faculty and trainees research methods is currently lacking, low-certainty evidence suggests that e-learning may be at least as effective as traditional learning for healthcare professionals; however, this research focuses on health professionals' behaviours, skills and knowledge as related to patient care and outcomes [28, 29].

Developing online educational modules for highly sought-after systematic review training will create time-efficiencies in providing library support while also broadening the coverage of the training available. In making this educational content openly available, we hope to extend these efficiencies to other academic and hospital libraries and to help address researcher training gaps at these institutions. We plan to license the online educational modules under a Creative Commons Attribution-ShareAlike 4.0 International License, allowing other libraries to remix, transform, or build on the material as they see fit for their researchers and institutions. After the educational modules are built, piloted, and deployed at the authors’ institution, the content will require on-going updates to stay current with new evidence and guidance. When the online education modules are made freely available and shared, we will welcome libraries to provide feedback about the content or delivery that may be considered for future updates and improvements.

This study examined systematic review services at libraries that support medical schools in Ontario and may not necessarily be representative of other libraries. The responses were a combination of retrospective self-report (e.g., proportion of time spent on tasks) and objective reported measures (e.g., number of supported reviews in a given year) by library respondents. While this study specifically investigated perceived needs for systematic review support at Ontario medical schools, future research could examine the needs of other disciplines and types of syntheses.

This study elucidates the need for more extensive systematic review support at Ontario medical schools and their affiliated hospitals; support that goes beyond the foundational steps of the review process that most libraries reported providing, including developing a research question, search strategy, and screening. To support medical and other health sciences researchers in producing high quality systematic reviews, institutions need to seriously consider how support can be provided for the later steps of the review process such as data extraction, analysis, and presentation of results. Online educational materials that introduce all stages of the systematic review process cannot replace the value that direct support from librarians, biostatisticians or methodology experts can provide. Making a freely available, online education module series that libraries and institutions could recommend to researchers may, however, offer a more efficient way to familiarize researchers and trainees with best practices and universally accepted reporting guidelines for performing a high-quality review.

## Supplementary Material

Online Supplement file

## Data Availability

The de-identified data generated and analyzed during the current study will be openly available in the Scholars Portal Dataverse repository, licensed under a Creative Commons Attribution-ShareAlike 4.0 International License, until December 2025 [10.5683/SP2/29IAYJ].
