# Supplementary material for: Systematic review support received and needed by researchers: a survey of libraries supporting Ontario medical schools
Source: J Can Health Libr Assoc. 2021 Dec 1;42(3):154–63. doi: 10.29173/jchla29571 (PMC9327595; doi:10.29173/jchla29571)
Supplement: Supplementary file 1 — Online Supplement file [file JCHLA-42-154-s001.pdf]

Needs of Ontario health sciences libraries supporting systematic reviews

Please Note: Survey questions intended for each library as a whole (not individual librarians). *Please use the last two academic years (or equivalent) as the benchmark for answering the below questions.

Information Letter
Study Title: Needs Assessment of Ontario Libraries for Supporting Systematic Reviews
Name of Principal Investigator: Sandra McKeown, Health Sciences Library, Queen's University
Name of Co-Principal Investigator(s): Zuhaib Mir, Eleftherios Soleas and Jennifer Ritonja

I am inviting library staff in Ontario academic and health institutions to take part in a research study. The purpose of this study is to see what the needs are for a systematic review workshop taking place in the online setting and being freely available. If you agree to take part, you will be asked to complete an on-line survey. The survey should take you about 10-15 minutes to complete. There are no known risks and the questions are about the provision of library services and support. There are no direct benefits or compensation to you as a participant. Study results will help add to the body of literature about the support of systematic reviews at the library and institutional level. We plan to publish the results of this study in academic journals and present them at conferences.

Participation is voluntary. You don't have to answer any questions you don't want to. You can stop participating at any time without penalty. Participants' confidentiality will be protected to the extent possible as permitted by the applicable laws. You may withdraw from the study up until December 31st 2020 by contacting me at Sandra.McKeown@Queensu.ca. After this point data will be de-identified and isolating individual responses will no longer be identifiable.

In the survey you will be giving responses that identify your institution, but not you personally. I will keep your data securely for at least five years per Queen's University Policy, after which the de-identified data will be deleted and purged electronically. In addition to the Principal Investigator and study team, the Queen's University Health Sciences and Affiliated Teaching Hospitals Research Ethics Board (HSREB) may require access to your study-related records to monitor the ethical conduct of the research.

This Letter of Information provides you with the details to help you make an informed consent choice in the survey. Your answer to the consent choice question takes place in the survey. All your questions should be answered to your satisfaction before you decide whether or not to participate in this research study. You have not waived any legal rights by consenting to participate in this study.

If you have any questions about the research, please contact me at Sandra.Mckeown@queensu.ca or 613-533-6000 ext. 75284. This study has been reviewed for

ethical compliance by the Queen's University Health Sciences and Affiliated Teaching Hospitals Research Ethics Board. If you have any concerns about your rights as a research participant please contact – Dr. Albert Clark, Chair of the Queen's University Health Sciences and Affiliated Teaching Hospitals Research Ethics Board at 1-844-535-2988 or HSREB@queensu.ca.

Wishing you well, Sandra and the Research Team

Consent Do you consent to participate in this study?

- ○ Yes
- ○ No

Skip To: End of Survey If Do you consent to participate in this study? = No

---

Q1 What institution does your library primarily support?

____

Q2 What library setting do you work in?

- ☐ Hospital
- ☐ Academic
- ☐ Other (Please specify) ____

Q3 What training do your librarians receive with regards to systematic review (please select all that apply)

- ☐ Cochrane
- ☐ Joanna Briggs Institute
- ☐ Library and Information Sciences Graduate Degree Courses
- ☐ Communities of Practice/ Peer Learning Communities
- ☐ Other systematic review workshop (Please specify) ____
- ☐ Not applicable

Q4 What type of systematic review support does your library provide? (Please select all that apply)
Please use the last two academic years (or equivalent) as the benchmark for answering the below questions.

- ☐ Educational Support
- ☐ Collaboration/ Participation with Review Team
- ☐ Other (e.g., Library Website Content) ________________
- ☐ We do not support systematic reviews

Q5 Which user groups do you regularly support with systematic reviews?

- ☐ Faculty/Clinicians
- ☐ Staff (e.g., Research Staff, Educational Support and Consultants)
- ☐ Trainees/Learners (e.g., Residents, Undergraduate, Graduate, Professional, Post-Doctoral Fellows)

Q6 What are the disciplines of the user groups you regularly support with systematic reviews? (Please select all that apply)

- ☐ Medical
- ☐ Nursing
- ☐ Rehabilitation Therapy (Physiotherapy and Occupational Therapists)
- ☐ Pharmacy
- ☐ Life and Health Sciences
- ☐ Other Health Sciences (Please specify) ________________
- ☐ Non-Health Sciences (Please specify) ________________

Display This Question:

If Please use the last two academic years (or equivalent) as the benchmark for answering the below q... = Educational Support

And Please use the last two academic years (or equivalent) as the benchmark for answering the below q... = Collaboration/ Participation with Review Team

Q7 Please use the last two academic years (or equivalent) as the benchmark for answering the below questions.

What is the breakdown of the support that you provide? (Slider- Fixed Sum- Totals must add up to 100).
 ________ Educational Support
 ________ Collaboration/Participation with Review Team
 ________ Other (Please specify)

Display This Question:

If Please use the last two academic years (or equivalent) as the benchmark for answering the below q... = Educational Support

Q8 Please use the last two academic years (or equivalent) as the benchmark for answering the below questions.

When providing educational support, how much is in: (Slider- Fixed Sum- Totals must add up to 100)
 ________ The form of ad-hoc consultations (individual and group)
 ________ Through teaching activities like library workshops and courses

---

Display This Question:

If Please use the last two academic years (or equivalent) as the benchmark for answering the below q... = Collaboration/ Participation with Review Team

Q9 Please use the last two academic years (or equivalent) as the benchmark for answering the below questions.

When collaborating with or participating on a review team, how much is as: (Slider- Fixed Sum- Totals must add up to 100)

_______ As a co-author

_______ As a non-author

Q10 What formal systematic review training is available at your institution for researchers? (Please select all that apply)

- ☐ Formal workshop provided by my library
- ☐ Formal workshop provided by my institution
- ☐ My institution refers folks elsewhere for review training (please specify where): ____
- ☐ No formal workshop/guidance at my institution

Display This Question:

If What formal systematic review training is available at your institution for researchers? (Please... = Formal workshop provided by my library

Q11 Please provide more details about the formal workshop(s) provided by **your library**.

- ○ Online or in-person? ____
- ○ Duration of workshop ______________________
- ○ Cost or free-for-user ____
- ○ Number of times offered per calendar year __________________________

Display This Question:

If What formal systematic review training is available at your institution for researchers? (Please... = Formal workshop provided by my institution

Q12 Please provide more details about the formal workshop(s) provided by **your institution**.

- Online or in-person? ________________
- Duration of workshop
____
- Cost or free-for-user _________________
- Number of times offered per calendar year_______________________________

Q13 How many systematic review projects has **your library** supported, in any capacity (educational support or collaboration/participation on review teams), in the following years (best guess)?

- 2017 ____
- 2018 ____
- 2019 ____
____
- So far in 2020 ____

Q14 How useful would **your library** find a freely available, online educational module series for all stages of the systematic review process?

- Not at all useful
- Not very useful
- Somewhat useful
- Very useful
- Extremely useful

Q15 How likely would **your library** be to recommend/incorporate the online educational module series into current support for researchers conducting systematic reviews?

- ◯ Not at all likely
- ◯ Not very likely
- ◯ Somewhat likely
- ◯ Very likely
- ◯ Extremely likely

Q16 What considerations did you take into account when answering Questions 14 and 15?

____

____

____

Q17 What aspect(s) of conducting systematic reviews do you feel **your end-users** require the most support? (Please select all that apply)

- ☐ Developing the research question
- ☐ Search strategy
- ☐ Screening
- ☐ Data extraction
- ☐ Analysis
- ☐ Presentation of Results

Q18 Are there any gaps in the systematic review support that **<u>your library/institution</u>** provides? (Please select all that apply)

- ☐ Developing the research question
- ☐ Search strategy
- ☐ Screening
- ☐ Data extraction
- ☐ Analysis
- ☐ Presentation of Results

Q19 Are there other libraries in your region that support researchers conducting systematic reviews (e.g. affiliated teaching hospital)?

- ○ Yes (Please specify) ____
- ○ No
- ○ Unsure

End of Block: Default Question Block
